# The role of morphology in novel word learning: a registered report

**DOI:** 10.1098/rsos.230094

**Published:** 2024-06-26

**Authors:** Olga Solaja, Davide Crepaldi

**Affiliations:** ^1^ Scuola Internazionale Superiore di Studi Avanzati (SISSA), Trieste, Italy

**Keywords:** word learning, morphology, reading, eye movements

## Abstract

The majority of the new words that we learn every day as adults are morphologically complex; yet, we do not know much about the role of morphology in novel word learning. In this study, we tackle this issue by comparing the learning of: (i) suffixed novel words (e.g. *flibness*); (ii) novel words that end in non-morphological, but frequent letter chunks (e.g. *fliban*); and (iii) novel words with non-morphological, low-frequency endings (e.g. *flibov*). Words are learned incidentally through sentence reading, while the participants’ eye movements are monitored. We show that morphology has a facilitatory role compared with the other two types of novel words, both during learning and in a post-learning recognition memory task. We also showed that participants attributed meaning to word parts (if *flibness* is a state of happiness, then *flib* must mean happy), but this process was not specifically triggered by the presence of a suffix (*flib* must also mean happy in *fliban* and *flibov*), thus suggesting that the brain tends to assume similar meanings for similar words and word parts.

## 1. Introduction

People encounter new, previously unknown words on a daily basis. In order to preserve successful communication, these new words must be interpreted quickly, essentially online. This is one of the mechanisms whereby adults expand their vocabulary throughout their lifetime [1–3]. People encounter most new words while reading, which implies that learning happens mostly implicitly, in the absence of any instruction or explanation. This suggests that people are able to compute meaning for novel words online during reading, by relying only on the information provided in the text [4].

Understanding an unknown word is mostly supported by the context in which the novel word appears, i.e. the surrounding words [5]. For example, in a sentence like ‘John was incredibly hungry, so he headed for the kitchen and had all the *wugs* that were left over from dinner’, it is not difficult to gather that *wugs* are some kind of food. Upon multiple encounters, especially across different conceptual domains [6,7], people reliably attribute meaning to the novel lexical item.

Some information about word meaning can also come from the word’s form itself, however. As symbolic systems, human lexicons are largely arbitrary; so, generally speaking, we would typically not be able to guess the meaning of a word based on how it looks, or sounds. However, regularities in the mapping between form and meaning do exist [8], and they seem to affect lexical processing [9–12].

Form and meaning maximally correlate through morphology. The words *gardener*, *seller*, *influencer* and *driver* all indicate a profession, and they seem to do so by virtue of their ending, -*er*. Similarly, the words *grasp*, *graspable*, *grasping* and *ungrasp* share a common core, which is related to their stem, *grasp*-. Even though the form-meaning mapping brought about by morphology is not always perfectly straightforward (e.g. *corner* is not someone who corns, and *irony* is not made of iron), morphology does establish some regularity in the way that words’ form is connected to words’ meaning. In this paper, we focus on this source of information in the novel words and investigate the effect of suffixes on the acquisition of new lexical items.

Morphology is widespread in human lexicons [13–16] and polymorphemic words account for most novel words that enter the lexicon [17]. As such, it is a primary player in word identification, particularly in the visual modality ([13,18–21], for a review, see [22]). This holds true for essentially all the languages that have been studied in the literature we describe here (e.g. English, French, German, Italian and Spanish), which all seem to exhibit quite similar mechanisms. For example, response times in lexical decision are proportional to the frequency of the stem of the target word [22], and the visual word identification of a stem (e.g. *depart*) is speeded by the previous presentation of a morphological relative (e.g. *departure*) in a way that cannot be traced back to the semantic and orthographic similarity between the two words [23,24]. It is now completely undisputed that words’ morphological structure is engaged during lexical processing.

More critically for the present work, these morphological effects are not limited to well-known, familiar lexical material. In fact, morphology had made its way to the psycholinguistic stage when Taft & Forster [25] discovered that non-words embedding existing stems (e.g. *de-juvenate*) are more difficult to reject in a lexical decision task than non-morphological controls with non-existing stems (e.g. *de-pertoire*). Since this seminal work, there have been many reports of morpheme interference effects in non-words [26–29] and non-word morphological priming [30–33]. This clearly shows that morphemes are addressed in unfamiliar letter strings, which is of course a necessary condition for word learning to be affected by the morphological structure of words. However, it is not entirely clear whether this information—which this work shows to be available to the readers’ cognitive system—is effectively used during word learning; in none of these experiments, in fact, were the unfamiliar stimuli learned as potentially meaningful novel lexical items.

This was the case, instead, in Tamminen *et al.* [34]. In a series of word-learning experiments, participants were familiarized with novel words made up of an existing stem and a new suffix (e.g. *crabafe*). For each of these novel words, a definition was created by using the meaning of each novel affix consistently, to modify the meaning of each familiar stem. For example, *crabafe* would be the zoo building where you can see exotic crab species and *gunafe* the section of an armoury where one can find a gun; in these examples, *-afe* refers to a place, similarly to *-ery* in *bakery* and *nunnery*. Participants were presented with the novel words and their definitions, and were then asked to type the word back. In a separate task, they were asked to recollect the word upon hearing its definition. Based on this training, participants were able to extract the suffix meaning, and to generalize the newly acquired knowledge to untrained novel words after a memory consolidation period of 7 days. For example, they read aloud more quickly *sailafe*, an item to which they were never exposed during training, when a preceding sentence context was consistent with the locative meaning of *-afe*. Interestingly, learning came up considerably more quickly (e.g. right after training) and with lesser constraints (e.g. without a need for high contextual diversity) in tasks that required deliberate reasoning, thus showing a dissociation between implicit and explicit memory. We will address this issue more in depth below. Again on the explicit dichotomy versus implicit dichotomy, but this time with regard to the training routine, participants were explicitly instructed to learn the novel words, but no explicit information was given about the suffixes, whose existence was left for the participants to figure out. The morphological training was thus entirely implicit.

In Havas *et al*. [35], participants were Finnish and Spanish speakers who were exposed to novel word–picture pairs. The novel words consisted of a non-word stem (*elu-*) and a novel suffix that indicated gender (*elu-ri*). The novel words were paired with pictures that depicted animals wearing typical male or female clothing. Participants were asked to learn the novel word–picture correspondences, but were not informed about the morphological structure—similarly to Tamminen *et al*. [34], there was no explicit morphological training. The recognition memory and rule generalization tasks showed, respectively, that participants were able to successfully recognize the items from the training among the distractors, and also to generalize the novel gender-marking system to new stems. In the generalization task, participants were presented with a new picture of an animal in male or female clothes, paired with two letter strings: both contained a stem that was not seen in the training, paired with either the feminine or the masculine suffix. In this study, there was no period of consolidation, suggesting that participants were able to use the newly acquired morphological knowledge straight away.

More recently, Dawson *et al.* [36] examined whether developing readers learn novel words better when these words contain suffixes. More specifically, they compared novel words whose meaning was congruent with the dominant meaning of a suffix in the language (e.g. *brint-ise*, to make an object clean again), against novel words that were still suffixed, but with a meaning that was not congruent with the meaning of the suffix in the real language (e.g. *drict-ful*, to put something in fancy dress). They found that congruency facilitated the learning of the meaning of the novel words, but this advantage did not extend to the phonological, orthographic or lexical level.

These findings suggest that humans extract morphological information while learning the meaning of unknown words. Moreover, they do so in the absence of any explicit instruction regarding morphology—in none of the studies above were participants cued to the presence of suffixes, or to their contribution to the form and meaning of the novel words. On the other hand, though, an important part of morphology-based learning in real life is based on familiar suffixes attached to unfamiliar stems, e.g. one might learn what *glare* means based on hearing the word in an informative context. Tamminen *et al*. [34] focused exclusively on the opposite case, that of an unfamiliar suffix attached to a familiar stem. Havas *et al*. [35] did use unfamiliar stems, but the focus was on the generalization of the morphological rule based on the novel suffixes; the learning of the novel stem was not tested. In a sense, these studies focus on how we learn morphemes, while the present work will focus instead on how morphemes affect learning. In this sense, we are closer to Dawson *et al*. [36], which, however, did not test whether there was any meaning attribution to the novel stems, that is, they did not assess stem learning. Moreover, and perhaps more importantly, participants in these studies received extensive and explicit training on the novel words themselves, which differs substantially from how word learning mostly happens in real life—implicitly, without instructions or explicit feedback.

A step toward a more ecological training regime was taken by Ginestet *et al*. [37], who followed previous studies with eye tracking [6,38] and trained their participants by embedding novel words into short stories. Of relevance for this paper, the novel words could contain an existing prefix and an existing suffix (e.g. *re-lurb-er*) or non-morphological chunks (e.g. *pe-lurb-le*). Similarly to previous studies [38], Ginestet *et al*. tracked the learning pattern during repeated exposures via eye tracking metrics. In general, they found that non-complex words attracted more fixations than complex words. It was not clear, however, whether there was a difference in looking times; there was a significant effect of word type on gaze duration, but not on the duration of single fixations, first-of-two fixations and second-of-two fixations. The learning pathway through repeated exposures did not change according to the morphological status of the novel words, when this was measured via the number of fixations or the duration of first-of-two fixations; however, it did change when considering gaze duration or total duration. The overall picture was a bit unclear across different eye tracking measures. There was, however, an interesting pattern in gaze duration: while non-morphological words required longer looking times at the beginning of the training, the difference disappeared, or at least shrunk, after four encounters with the novel words. In terms of post-training accuracy, complex words were spelled more accurately than orthographic controls.

In the present work, we build on and extend Ginestet *et al*. [37] in two main ways. Firstly, we will connect with a large branch of the recent morphological literature, which focuses on the role of meaning-bearing *per se* versus the role of letter co-occurrence statistics. This was triggered by the finding that a pseudo-suffixed word like *corner* speeds up the processing of its pseudo-stem *corn* in the primed lexical decision to a similar extent as a genuinely suffixed prime (e.g. *farmer-farm*) [39,40]. More recently, it has been shown that chunks of pseudo-letters, with no connection with phonology or semantics, can mimic some classic morphological effects [41]. This further underlines the strong effect that the mere frequency of a letter cluster can yield. In the context of the present work, one might imagine that novel words with existing suffixes are learned more easily because they contain meaningful elements, or because they feature frequent letter chunks. Thus, we will contrast: (i) novel words that contain a suffix to items that contain an ending of the same frequency, but do not have any meaning; and (ii) novel words with high-frequency endings to items with endings that are lower in frequency. Such a design will rather cleanly separate meaning-based morphological effects from frequency-based effects.

Secondly, in addition to focusing on orthographic learning, we will also investigate the extraction of the meaning of the novel stem—we will ask whether participants learn that, if *flibness* is a state of happiness, then *flib* must mean happy.

A further important feature of the experiment we propose is that we will investigate the outcome of learning both implicitly and explicitly. We mentioned above evidence that explicit representations of word meanings can develop rapidly, but implicit representations take more time and/or exposure. This notion is supported by several studies. In an experiment by Batterink & Neville [42], participants were exposed to pseudo-words embedded in a narrative. Afterwards, they were administered behavioural tasks while event-related potentials (ERPs) were recorded. The results showed that in a primed lexical decision task, which measured learning implicitly, there was no effect of priming, either in behaviour or in electrophysiology. On the other hand, in the recognition task (explicit measure), a robust N400 effect was found for the correctly learned word. Exploring the timeline of the development of implicit word representations, Qiao & Forster [43] taught participants novel words that were designed as neighbours of existing words (e.g. *bontract*, a neighbour to *contract*). They were tested in a primed lexical decision task. The prediction was that if the novel word is successfully learned, it will yield inhibition, as it is typically found for pairs like *contract-CONTRAST*) [44–46]. The results showed that inhibition only emerged after four sessions of training spread over four weeks. This evidence sits nicely with general theories of learning and memory that postulate the slow integration of novel information into a highly interconnected system of overlapping memories [47–50]. In such a system, new information must be acquired and integrated at a slow pace, in order to avoid interference with pre-existing knowledge. This slowness of learning also allows the system to capture structural, persistent aspects of the input (e.g. a new word occurring across a set of different sentences), rather than volatile details or irrelevant information (e.g. whether the same word is pronounced by a male or a female speaker). Within this framework, explicit and implicit memory subserve fundamentally different goals, and are structurally different; the former is fast, does not lead to integration with existing knowledge and decays relatively quickly; the former is slow, but embeds into a highly sophisticated system of existing memories and is more resistant to decay. Notice that, as general statements about learning and memory, these considerations do not speak directly on word learning, and even less on the role of morphology in the process. Thus, there are no direct predictions that emerge here and that this study wants to test. Rather, these general theories provide the broader landscape for the investigation of word learning, and the psycholinguistic data and theorizing described above beg the question of how morphology plays out here, given the deep role that it plays in word processing and the scant evidence we have collected thus far.

In the present experiment, eye tracking will provide an implicit measure of learning, where we will monitor the reduction of looking times across progressive encounters with the novel words. We will use the recognition memory task as an explicit measure. In addition, we will also investigate both the implicit and the explicit extraction of the stem meaning—via a sentence congruency task and a definition selection task, respectively.

In what follows, we first describe the experimental design and paradigm; then we present a pilot study that we have conducted with 14 participants; and finally, we illustrate how the pilot helped us fine-tune some of the experiment parameters and adapt the design to the outcome of a power analysis.

## 2. Methods

### 2.1. Novel words

Eighteen novel stems were created as readable combinations of letters that do not exist as stems or words in Italian (e.g. *pobed-, cribot-*). They were five or six characters in length; their mean log bigram frequency was 5.77 (s.d. = 0.31); their mean average Levenshtein distance to the 20 closest lexical neighbours (OLD20 [51]) was 2.13 (s.d. = 0.27); and they had no immediate lexical neighbour (their edit distance to the closest word was greater than 1). These 18 stems were sorted into six triplets, so that the members of each triplet would be rotated through three main experimental conditions (see next paragraph). Within each triplet, stems were of the same length, and matched as closely as possible for log bigram frequency (5.72 [s.d. = 0.25] versus 5.66 [s.d. = 0.41] versus 5.91 [s.d. = 0.25]) and OLD20 (2.25 [s.d. = 0.34] versus 2.13 [s.d. = 0.31] versus 2 [s.d. = 0]).

To generate the novel words to be learned in the experiment, the stems were paired with three types of endings: (i) suffixes (e.g. *-enza*; a corresponding example in English would be *-ness*); (ii) non-morphological endings matched in frequency to suffixes (5.01 [s.d. = 0.24] versus 4.73 [s.d. = 0.36]; e.g. *-ondo*; a corresponding example in English would be *-an*); and (iii) non-morphological ending lower in frequency than suffixes (2.37 [s.d. = 0.20] versus 4.73 [s.d. = 0.36]; e.g. *-espa*; a corresponding example in English would be *-ov*). Frequency was specific to the word final position [28]. The stimuli were selected from [52]. As mentioned above, we rotated the 18 stems across the three types of endings in a classic Latin square design; therefore, the overall stimulus set was composed of 18 × 3 = 54 novel words, but each participant only learned 18 (i.e. for each participant, each stem was paired with only one given ending, with six endings per condition). Overall, the novel words had a mean log bigram frequency of 6.01 (s.d. = 0.19) and a mean OLD20 of 3.78 (s.d. = 0.36). None of the words had any immediate lexical neighbour.

### 2.2. Learning task

Each target word was embedded in 10 different sentences (e.g. ‘Fare il *cribotista* non porta tanti soldi, però sai che fai bene all’ambiente e agli animali’, ‘You don’t make much money working as a *cribotista*, but you help the environment and the animals’). Thus, each participant will read 180 sentences in total, and will be exposed to each novel word 10 times.

Sentences were constructed to convey the meaning of the novel word; for a couple of illustrative examples, see table 1 (the full stimulus set is available at https://osf.io/x7ctg/). We tried to use fairly diverse contexts (see [6,7,53]), and to give sentences similar syntactic structures. The novel words never appear in the first or last position. The training sentences contain 16 words on average (s.d. = 1.57; range: 13–19) and will be presented one by one in the centre of the screen. Participants were asked to press the spacebar when they are ready to read a new sentence. They were instructed to read sentences and try to understand them even if there are some unknown words. The order with which the sentences were presented was randomized across participants.

**Table 1.** Example of two novel words each used in three different sentences. Original followed by a translation into English.

target word	training sentence
rugobenza	Marco non ha *rugobenza*, quindi quando la madre lo ha sgridato, si è messo a urlare anche lui.
	Marco doesn’t have any *rugobenza* so when his mother scolded him, he also started to yell.
	Quando ho rotto la bottiglia dell'olio, ho dovuto ascoltare con *rugobenza* il rimproverò di papà.
	When I broke a bottle with oil, I had to listen to dad’s scolding with *rugobenza*.
	Trent'anni fa i bambini dovevano sopportare con *rugobenza* e senza rispondere i rimproveri della maestra.
	Thirty years ago, children had to endure teacher’s criticism with *rugobenza* and in quiet.
cribotista	Alessandro lavora da tanto come *cribotista*, ormai è famoso per le sue capanne sotto terra.
	Alessandro has been working as a *cribotista* for a long time now, he’s famous for his underground huts.
	Nel neolitico, il *cribotista* era molto importante per la tribù perché construiva i ripari per tutti.
	*Cribotista* was very important for a tribe in the Neolithic because they built shelter for everybody.
	Per fare il *cribotista*, bisogna intendersi della natura e soprattutto del terreno e delle acque sotterranee.
	To work as a *cribotista*, you have to know a lot about nature, and terrain and groundwater above all.

### 2.3. Testing tasks

#### 2.3.1. Recognition memory

Participants were asked to identify each trained novel word in a recognition memory task. Similarly to the approach of [34], there were three types of distractors: (i) untrained stem + trained ending (e.g. *bepolenza*; an English equivalent would be *tarpness*); (ii) trained stem + untrained ending (e.g. *rugobiera; flibist*); and (iii) trained stem + trained ending, but in new, unseen combinations (e.g. *rugobondo; flibable*). Each item was presented once, for a total of 60 trials; 18 trained items, 18 recombinant items and 12 items for each of the other two distractor types. Clearly, the trained and recombinant items depend on the rotation a participant was assigned to in the learning phase; thus, we created three matching rotations in this task as well.

Items appeared one at a time at the centre of the screen, and participants were asked to decide whether they remember the item from the training via a button press on the keyboard. Items stayed on the screen until participants respond.

#### 2.3.2. Sentence congruency

The sentence congruency task was designed to assess whether participants assign meaning to the stems of the novel words they learned. For example, the novel noun *rugobenza* contains the familiar, existing suffix *-enza*, which implies that *rugob-* is a novel stem. The training sentences provided meaning to the novel words, and therefore to their novel stems (e.g. *rugobenza* refers to ‘being able to stand someone yelling at you without overreacting’, and thus the stem *rugob-* must have something to do with the ability to accept scolding without much complaining). Free stems do not exist in Italian,^1^ at least for content words, so we have to use what we call *base words*, i.e. words where the novel stems are attached to the unmarked morphological suffix (e.g. *rugob-are*, an infinitive verb; *cribot-ista*, a singular noun). It was not easy to anticipate if readers would infer the existence of a novel ‘stem’ when the trained word does *not* contain a suffix, i.e. in the non-morphological ending conditions. In fact, in such cases there is not really a stem at all; and if, as one might intuitively think, the learning of a stem is triggered by the presence of a familiar suffix, there should be no stem learning in these conditions.

For each base word, two sentences were constructed, which are either consistent or inconsistent with the meaning of the base word itself (e.g. congruent meaning: ‘Quando la mamma mi ha sgridato, abbiamo litigato di brutto, non ce l'ho fatta a rugobare’—‘When my mother yelled at me, we had a big row, I was incapable of *rugobare*’; incongruent meaning: ‘Quando la mamma mi ha sgridato, era troppo tardi, non ce l'ho fatta a rugobare’—‘When my mother yelled at me, it was too late, I was incapable of *rugobare*’). The critical base word was always at the end of the sentence in both sets. Although there is some debate as to whether the sentence final position has a special role in handling inconsistencies that were not resolved within a sentence [54], our primary goal here was being able to focus on an area that is most likely to reflect integration processes. Sentences in the two sets had very similar structures. They were divided into two rotations, which were counterbalanced across participants, so that no single participant saw corresponding sentences in both congruent and incongruent conditions. Sentences appeared one at a time on the screen while the eye movements were recorded. Each sentence was followed by the question ‘Does this sentence make sense?’, to which participants replied with a button press on a keyboard. There was no time out, neither for sentence reading nor for the comprehension questions.

#### 2.3.3. Definition selection

Participants were presented with the 18 base words corresponding to the 18 novel words that they had learned, and had to choose among four definitions. Besides the correct option (e.g. ‘standing someone yelling at you without overreacting’ for *rugobenza*), the alternatives were: (i) related, but underspecified (e.g. ‘to be patient’); (ii) unrelated (e.g. ‘to listen to someone’s stupid comments’); and (iii) the definition of another base word (e.g. ‘distrust in gossip’, which is the definition for another novel word in the experiment, *zudulare*). This latter option allowed us to make sure that participants did not simply choose some semantic content that they were exposed to during the experiment, but specifically remember the correct link between form and meaning. Trial order was randomized across participants. Each item appeared in red on the top part of the screen. The four possible definitions were numbered and displayed one under the other. Participants selected their choice by pressing the appropriate number on the keyboard (1, 2, 3 or 4). There was no time out.

### 2.4. Sensitivity to morphemes

Prior to the experiment, we probed participants’ sensitivity to the morphological structure of non-words using a morpheme interference task. The task uses a lexical decision paradigm where the critical comparison is between the rejection time for morphologically structured non-word (e.g. *fruitness*) and orthographic controls (e.g. *fruitnuss*), and it has been extensively used in the morphological literature [25,28,55–57], although not as a measure for individual variability. Recently, De Rosa & Crepaldi [52] developed a version of this task with Italian materials; we used that task here. The critical conditions (morphological and control, orthographic non-words) contained 60 items each, which differ by one letter (e.g. *lesionaggio* versus *lesioneggio*). Stimuli in these conditions were matched for the number of orthographic neighbours (N) (0.25 [s.d. = 0.44] versus 0.05 [s.d. = 0.22]), OLD20 (2.66 [s.d. = 0.43] versus 3.02 [s.d. = 0.48]) and length (8.97 [s.d. = 1.19] versus 8.97 [s.d. = 1.19]). Of course, the task also included 48-word stimuli as fillers (i.e. YES trials), 24 of which were complex (N = 1.83 [s.d. = 1.09], OLD20 = 2.40 [s.d. = 0.49], length: 8.71 [s.d. = 1.04]) and 24 of which were not (N = 1.58 [s.d. = 1.14], OLD20 = 2.48 [s.d. = 0.45], length: 8.79 [s.d. = 1.14]). To ensure that no single participant was exposed to both the complex and non-complex version of the same critical item (e.g. *fruitness* and *fruitnuss*), the task included two rotations that were counterbalanced across participants; so, each participant was presented with 30 complex non-words, 30 control, orthographic non-words, 24 complex words and 24 simple words. Item presentation was randomized across participants. Items were presented one at a time at the centre of the screen until keyboard response.

### 2.5. Apparatus and software

The experiment was programmed in Python (v. 3.6.6; [58]), using Psychopy (v. 1.11.0.0; [59]) and Pylink (v. 2020.2.3; [60]). Stimuli were presented in white against a black background, using the Courier New font (size 25). They were presented on a 27-inch monitor, at a viewing distance of 62 cm. The refresh rate and resolution of the monitor were 144 Hz and 1920 pixels × 1080 pixels, respectively. To record eye movements, we used an Eyelink 1000 Plus (tower mount; [61]). We recorded from the right eye, with a sampling rate of 1000 Hz. In order to minimize head movements, participants were asked to use a chin rest. Responses were collected via a keyboard.

### 2.6. Procedure

Participants first completed the morpheme interference task, which lasts approximately 10 min. They then moved on with the learning task (approx. 30 min), recognition memory task (approx. 10 min), sentence congruency task (approx. 20 min) and definition selection task (approx. 10 min). Participants were encouraged to take a break between the tasks. The whole procedure lasted approximately 1 h 30 min.

### 2.7. Data modelling and statistical analysis

Data were modelled and analysed in R [62]. For the computation of eye tracking measures, we used the Eyekit package for Python, v. 0.3.10 [63]. To model the data, we fitted (generalized) linear mixed-effects models as implemented in the *lme4* package [64]. We implemented treatment coding in all models. For visual inspection of the data distribution, we used the Box–Cox plot as implemented in the *MASS* package [65]; data were either logarithmically or inverse transformed to obtain the relevant distribution more Gaussian. All models included random intercepts for subject and stimulus, and the maximal random slopes structure as appropriate for the experimental design. In the case of convergence issues, we gradually simplified the random slope structure by removing (i) the interactions; (ii) then the random slope correlations; and finally (iii) the random slope for the main effects. After modelling the data, we assessed statistical significance using ANOVA from the *car* package [66]. To interpret the statistical patterns, we used both the estimated beta parameters in the model and the reconstruction of the expected response times/looking times/accuracies per condition, as computed by functions in the package *Effects* [66].^2^

### 2.8. Exclusion criteria

Individual data points affected by technical errors (e.g. we displayed the wrong word, the program deviated from the standard trial timeline) were planned to be discarded. If the prevalence of these technical errors is high (greater than 20%) for a given participant, or for a given item, we planned to reject the entire set of data referring to that participant or item. The eye tracking data were cleaned following a standard approach in the field. Entire trials were planned to be rejected when they were heavily affected by head movement, blinking or drift. Individual fixations under 80 ms were also discarded, along with excessively long fixations; the precise threshold here was defined after looking at the distribution of the data points. In the sentence congruency and the definition selection tasks (i.e. those tasks assessing the generalization of the meaning to the stem), we also excluded data from participants whose *d*-prime in the recognition memory task will be below 1; such a low *d*-prime would indicate that these participants did not learn the novel words in the first place, so it does not make sense to ask whether they learned any meaning for the stems.

## 3. Pilot study

We carried out a small-scale pilot study with the goal of putting our paradigm and experimental design to the test. We wanted to check whether people do indeed learn the novel words, and whether the way we operationalized our theoretical questions holds the promise to answer those questions, once a larger sample of data will be collected in the main study. In line with these goals, we limited ourselves to explore the pilot data and model them to estimate effect sizes that might inform a power analysis (of course, with consideration of the fact that effect sizes might deviate from the real, population effect size in a small-scale pilot).

### 3.1. Participants

Fourteen adults (three female; mean age = 28.6 years, s.d. = 3.7 years) took part in the pilot experiment. They were all native speakers of Italian, with normal or corrected-to-normal vision and no reading disabilities. Participants were paid 20 Euros for their participation.

### 3.2. Results

#### 3.2.1. Learning task

The novel words were fixated 5.29 times on average. The proportion of single fixations is relatively low, 24%, which is not surprising given that these were novel, unfamiliar items. The overall mean first fixation and gaze durations are 255 and 732 ms, respectively, and the correlation between these two metrics is 0.14 (which nicely reflects the low proportion of single fixations). Moreover, the target novel words were refixated quite often, 39% of the times.

Based on this initial overview of the data and on the relevant literature [6,67,68], we considered the following dependent variables: (i) the duration of the first fixation into the novel word; (ii) the sum of the duration of all fixations on the novel word during the first pass (i.e. until the gaze moves away from the word for the first time; *gaze duration*); (iii) the sum of the duration of all fixations on the novel word from the first fixation until the gaze moves past to the right (i.e. including any regression to preceding words on the left; *go-past time*); (iv) the sum of the duration of all fixations on the novel word (*total duration*); and (v) the probability of regressing into the target, after having gone past it to the right. This set of variables encompasses early to late processing.

**Figure 1.**
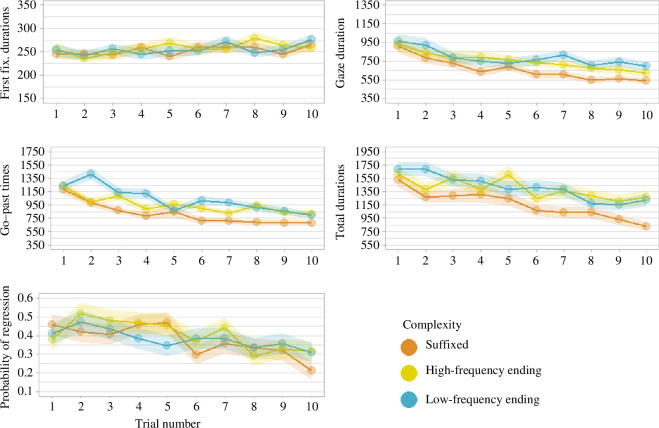
Eye tracking measures as they changed during the experiment, across the three main conditions. The shadowed areas represent the standard error of the mean.

Figure 1 shows how the eye tracking metrics change from the first to the last encounter with the novel words. Gaze duration, go-past time, total fixation time and probability of regression into the target word all seem to decrease with more encounters with the novel words. First fixations remain relatively flat, or even become longer. As to the comparison between the complexity conditions, it is not easy to observe patterns with such large confidence intervals (which is to be expected in a pilot experiment). Suffixed, high-frequency and low-frequency ending novel words appear to be very similar in the first fixation duration. Overall, the suffixed items seem to elicit shorter gaze durations, go-past times and total durations. The pattern is quite less clear as far as the probability of regression is concerned. Overall, it is difficult to see any potential sign of an interaction between condition and the way eye tracking metrics evolve over successive encounters with the novel words.

To explore these results further, we fitted a linear mixed model to each of the eye tracking measures, with low-frequency ending items as a baseline. Of course, this was not done with the aim of running statistical tests, but simply to provide some initial estimates for the effects of interest. Note that these estimates will unavoidably be quite imprecise in the current pilot, low-*N* study. In line with the considerations offered above, the *β* parameter for the number of encounters is different from 0 on all our variables: first fixation duration (*β =* 0.01, CI = [0.00 to 0.02]), gaze duration: *β =* −0.02, CI = [−0.03 to 0.00]), go-past time (*β =* −0.05, CI = [−0.07 to −0.04]), total durations (*β =* −0.05, CI = [−0.06 to −0.03]) and regressions back into target (*β =* −0.07, CI = [−0.12 to −0.01]). As for the complexity effects, modelling confirms what was observed from the raw data: this effect is virtually non-existent in the first fixation duration (high frequency (HF): *β =* 0.00, CI = [−0.07 to 0.08]; suffixed (SUFF): *β =* 0.01, CI = [−0.06 to 0.09], only a little more visible in gaze duration (HF: *β =* 0.04, CI = [−0.11 to 0.19]; SUFF: *β =* −0.02, CI = [−0.17 to 0.13]) and a bit more apparent in go-past time (HF: *β =* −0.18, CI = [−0.31 to −0.05]; SUFF: *β =* −0.24, CI = [−0.36 to −0.11]), total duration (HF: *β =* −0.09, CI = [−0.24 to 0.05]; SUFF: *β =* −0.17, CI = [−0.32 to −0.03]) and regressions (HF: *β =* 0.27, CI = [−0.24 to 0.77]; SUFF: *β =* 0.27, CI = [−0.23 to 0.77]).

Overall, these pilot data suggest that fixation durations might potentially change as the number of encounters with the novel words increases, in a way that is compatible with easier processing as a consequence of learning. Reduced number of regressions back into the novel words also seems to support this suggestion. Instead, we find no apparent sign of an interaction between number of encounters and condition.

#### 3.2.2. Recognition memory

The mean overall accuracy in this task was 0.81, suggesting that participants learned the novel words quite successfully. Recognition is much higher with suffixed words (0.90) than high-frequency (0.76) and low-frequency (0.76) items, although variance is quite high across participants (0.30, 0.43 and 0.43 in the three conditions, respectively) and therefore it is difficult to understand precisely how solid this difference might be. The mixed-effects model estimates the difference between suffixed and low-frequency items to be 1.16, CI = [0.04 to 2.28] in the log odds space.

As in any YES–NO recognition memory task, a good performance in the identification of familiar items can be achieved with a bias towards YES responses; at the limit, one might always respond YES, and this would guarantee a 100% performance in the identification of known items (hits), at the cost of a very high rate of false alarms (i.e. identifying as familiar items that are new instead). In addition, with the current design, there are more NO than YES expected responses (following the path set by previous studies, e.g. [34]), which might invite respondents to develop a bias towards NO answers. To control this potential confound, we computed a *d*-prime score for each participant, which corrects the performance on familiar items based on the rate of false alarms (*d*’ = *z*(hit rate) − *z*(false alarm rate)). The mean *d*-prime value across participants was 2.18, with a range of 1.33–3.91, showing that participants were indeed able to recognize novel words among distractors, both at the group level and all of them individually (there is no clear-cut threshold for reliable discrimination on the *d*-prime scale, but 1 is traditionally taken as indicating some ability to tease apart familiar from novel items [69,70]. Looking at the *d*-prime separately across conditions, we see that subjects were more sensitive to distinguish trained items from foils in the suffixed condition (*d*’ = 3.13), compared with the high-frequency ending (*d*’ = 2.15) and low-frequency ending conditions (*d*’ = 2.3). In addition, we checked the distribution of the *c* index, which tracks bias [71]. The median value was −0.10 (min: −0.60; max: 1.24). Thus, there is no sign that the higher number of expected NO responses elicited a widespread NO bias.

#### 3.2.3. Interim considerations

Overall, the eye tracking data in the learning task and the participants’ performance in recognition memory clearly suggest that the paradigm is effective in inducing some learning of the novel words. This learning also seems to interact nicely with our experimental manipulation. Of course, we cannot say whether the differences between conditions illustrated above are statistically reliable at this point, but they do suggest that the paradigm has the potential to reveal how word learning is affected by the presence of suffixes or high-frequency, non-morphological clusters.

One important note to underline here is that the data described thus far only indicate that participants gain familiarity with the novel words; they do not say, however, whether the readers also learned the meaning of these words, or whether they attributed meaning to the stems. This was the goal of the tasks that we are going to consider next.

#### 3.2.4. Sentence congruency

**Figure 2.**
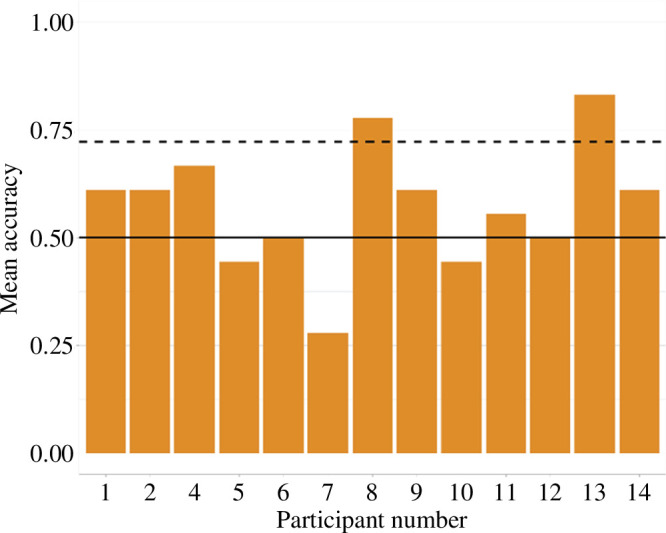
Accuracy across participants. The full line represents the chance level, while the dashed line represents the 95th percentile of a binomial distribution with *p*(correct) = 0.5 (chance level) and *n* = 18 (the number of trials that our participants undertook); that is, participants above this threshold are 5% or less likely to be responding randomly.

The overall accuracy in the behavioural part of this task, i.e. whether participants are able to distinguish congruent sentences versus incongruent sentences, was quite low, 0.57 on average. Breaking down by participants (see figure 2), only 2 out of 13 are 5% or significantly above chance, assuming a binomial distribution and guessing the probability of 50%. Interestingly, the accuracy rate seems to change quite little across the complexity conditions (SUFF: mean = 0.55, s.d. = 0.50; HF: mean = 0.60, s.d. = 0.49; low frequency (LF): mean = 0.56, s.d. = 0.50), which is quite revealing. If any stem meaning attribution might happen, this should show up specifically in the suffix condition, if it is primarily driven by the presence of a meaningful suffix; or in the suffix and the high-frequency ending conditions, if it is primarily driven by frequency. These data, instead, seem to suggest that there is little difference between the three conditions, and that stem meaning extraction did not happen—which is in line with the overall performance in this task, as illustrated at the beginning of this paragraph.

What do the eye tracking data suggest? Overall, the novel words were fixated 5.91 times on average. They were also refixated after rereading the previous part of the sentences in 67% of the trials. These figures suggest that participants were making an effort to assign a meaning to the novel base word, or at least integrate it somewhat into the broader semantic context provided by the sentence.

In addition to the metrics that we considered in the learning task (first fixation duration, gaze duration and total looking time), we examined here the summed durations of second-pass fixations and the probability of making a second pass. The critical word was always at the end of the sentence, and we reasoned that if participants are unsure about its meaning, they are likely to re-examine the sentence and then refixate the critical item. This behaviour would be captured most effectively by sum-of-second-pass and probability-of-second-pass.

**Figure 3.**
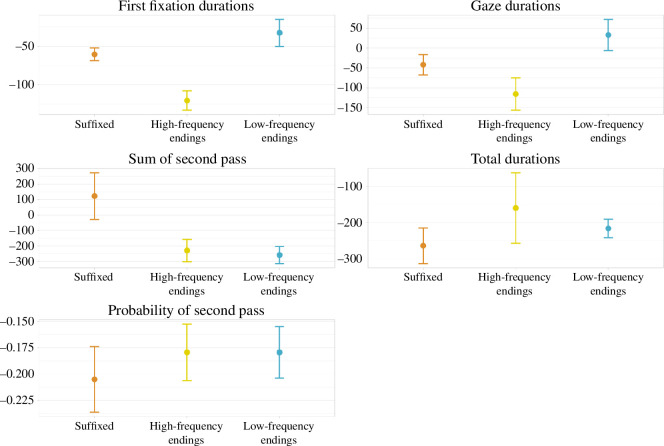
Congruency effect in the sentence congruency task across eye tracking measures and complexity conditions. Error bars represent the standard error of the mean.

The congruency effects on the different eye tracking metrics are represented in figure 3. First fixation and gaze duration show similar patterns; the congruency effect appears to be stronger with base words that come from novel words with high-frequency endings, as compared with low-frequency endings (*β* = −0.30, CI = [−0.57 to −0.03] and *β* = −0.14, CI = [−0.51 to 0.25], for first fixation and gaze duration, respectively) and suffixes (*β* = −0.15, CI = [−0.42 to 0.11] and *β* = −0.02, CI = [−0.42 to −0.37]). The probability of refixation is quite similar with base words from the three conditions (the model betas for the comparison across conditions are all very close to 0), which indicates that, even if first fixation and gaze duration might suggest better stem learning with high-frequency endings, all base words equally required extra processing. That is, whatever difference in learning across conditions is revealed by the first-pass metrics, it is probably not very solid. On the summed duration of second-pass fixations, the congruency effect estimates are larger with base words from the non-morphological conditions (*β* = −0.51, CI = [−1.06 to 0.04] and *β* = −0.41, CI = [−0.97 to 0.15] for low-frequency and high-frequency ending words, respectively, both contrasted with suffixed words). On total looking time, the pattern is reversed compared with first fixation and gaze duration: the congruency effect seems larger with base words from the suffix and low-frequency ending conditions, and smaller with base words from the high-frequency ending condition. However, the model betas for the cross-condition comparisons are close to 0 (low frequency versus high frequency: *β* = 0.01, CI = [−0.33 to 0.34]; suffixes versus high frequency:congruent: *β* = −0.05, CI = [−0.39 to 0.28]).

#### 3.2.5. Definition selection

The overall accuracy in this task was 0.69, indicating some ability to single out the correct definition of the base words among distractors. The performance was quite similar on base words coming from the three conditions; participants picked the correct definition 68% of the times for base words from the suffix condition, and 69% of the times for base words from the high-frequency ending and low-frequency ending conditions. The modelling obviously confirms that there is basically no difference between conditions (suffixed versus low-frequency endings: *β* = −0.07, CI = [−0.81 to 0.66]); high-frequency versus low-frequency endings: *β* = −0.00, CI = [−0.74 to 0.74]). Overall, this suggests that the meaning of the stem might be created adequately, and can generally be distinguished from meanings that are incorrect or not specific enough. However, this outcome does not seem to be influenced by the structure in which the stem was inserted and learned—whether this structure is morphological in nature, or at least contains word endings that are frequent in the language. Essentially, it seems that readers more generally build on any combination of orthographic and semantic information; when they learn that the novel word, e.g. *rugobenza*, means ‘being able to stand someone yelling at you without overreacting’, they tend to infer that a novel item that is orthographically similar to *rugobenza* might also mean something similar. It does not seem to matter very much whether the novel word is *rugobenza*, with a suffix that clearly flags the existence of the novel stem *rugob-*, rather than *rugobondo*, which does not feature any meaningful suffix or frequent ending that might suggest a breakdown of the novel word into separate units based on letter statistics [41].

#### 3.2.6. Sensitivity to morphemes

The average accuracy and response time in the morpheme interference task were 0.93 and 1390 ms, respectively. This latter figure is quite slow, which suggests that the participants in the pilot study might have missed our emphasis on speed; to fix this, in the main experiment we implemented a time out for response time in the practice phase of this task. The analysis of the data aggregated by item showed that two stimuli, *urtevole* and *flauteria*, were considered to be existing words by most participants, i.e. their accuracy was below 50%. They were substituted with two new items (*divaneria* and *untevole*) in the main task, in a way that does not affect the matching between the complex non-words and their orthographic control.

As expected, morphologically structured non-words were responded to less accurately (0.88) and more slowly (1734 ms) than their control, non-morphological controls (0.99 and 1557 ms, respectively). The effect size (calculated as the beta coefficient in the model) was estimated to be 55.17 (95% CI = 23.91 to 86.43) for the response times, and −2.61 (95% CI= −3.65 to −1.58) for accuracy.^3^ These figures nicely replicate previous literature [25,28,55], and thus attest to the reliability of the task.

Because we are primarily interested in individual variability here, an index of sensitivity to the morphological structure of non-words (morpheme interference index, MIF) was computed for each participant according to the following procedure. We subtracted the average on simple non-words from the average on complex non-words, separately for accuracy and response time. Since these two measures did not exhibit strong correlation (−0.18), we standardized them and summed them up. In the pilot sample, this index varies from −2.8 to 1.89 across participants (median = −0.23). We planned to use this index in an exploratory analysis in the main study, where we would correlate it with the accuracy and reading times measures in the tasks that we described above. In this way, we aim to explore whether individual sensitivity to morphology is associated with how one uses morphological information in the word-learning process.

#### 3.2.7. Debriefing

At the end of the whole testing session, we asked the participants whether they could guess the aim of the experiment, if they employed any specific strategies to complete the tasks, and how did they find the experiment overall. The aim was to gather information that might be useful to adjust the design of the experiment.

The participants’ responses indicate that they understood the task correctly. Unsurprisingly, several participants mentioned the morphological content in some items in the learning task; however, none of them guessed that this was a crucial aspect of the experiment. Moreover, participants did not report to have developed any specific strategy that would invalidate the outcome of the experiments. Most of them explained that they were simply trying to remember the words, while some reported that they tried to create a mental image of the concept. Finally, some participants reported that they looked for existing words in Italian that refer to similar concepts, to help memorize the novel words.

### 3.3. Final considerations

The eye tracking data in the learning task and the recognition memory performance show that the paradigm worked—participants seem to have learned the novel words. There was also a fairly clear effect of word ending on this learning, which was particularly prominent in the recognition memory task.

At the same time, however, the pilot data seem to indicate that this learning did not lead to any solid extraction of the meaning of the stems. The behavioural data in the sentence congruency task suggest that participants did not distinguish very clearly congruent from incongruent sentences containing the novel stems. The eye tracking data were a bit less clear, and possibly leave some room for the existence of a congruency effect showing up implicitly in eye movements. Yet, the congruency effects that timidly emerged in the different eye tracking metrics do not seem to form a coherent pattern.

The definition selection task offers evidence that participants were able to single out the correct definition of the base words; should this finding emerge in the main study, it would describe an interesting dissociation between the ability to process the base words while reading a sentence and the ability to figure out a definition for them, at least among the competing alternatives that we used.

Finally, there seems to be very little evidence overall that whatever was learned about the meaning of the stems, it came from a morphological analysis triggered by the presence of a familiar suffix, or from an analysis of the internal structure of the words that is informed by letter chunk frequency. The mechanism that seems to be in place is rather one whereby a more general correspondence between form and meaning is assumed on the part of the reader, so that whatever novel word begins with the same letter chunk, it must have a similar meaning—independently of morphology or frequency. Should this conclusion emerge in the main study, it would resonate nicely with theoretical accounts [72,73] and experimental data [8,11,12] that place morphology in the context of a more general attempt of the brain to find probabilistic ties between form and meaning of whatever nature. Under this view, morphology might not be a special domain of linguistic analysis that is qualitatively unique, and requires specific cognitive mechanisms and representations; rather, morphological sets would be ‘lexical islands’ where a reliable correspondence between form and meaning just emerges more clearly, but not in a way that clearly sets it apart from other types of more fuzzy, more probabilistic regularities in the correspondence between form and meaning.

### 3.4. Power analysis

#### 3.4.1. Methods

Since we planned to analyse our data via mixed-effect models, and there is no analytical treatment of power in this context as far as we are aware, we resorted to data simulation. We followed the approach described in [74], and generated 1000 datasets using the relevant effect sizes, and noise levels informed by previous experience in the laboratory and the pilot data described above. The code for this analysis is reported in the OSF repository for the project.

More specifically, for the learning and recognition memory tasks, we targeted two-thirds of the effect size that we observed in the pilot experiment, to account for a possible overinflation of these effects (as substantial deviations in the observed effect size from the theoretical population means are likely in small-scale pilot studies). It was more complex to understand which is the most proper approach for the sentence congruency and definition selection tasks. In fact, the effects that emerged there were very small, which would require a practically unfeasible sample size, if we were to reach a sufficient power level. In addition, if the effects are truly as small as the pilot data would suggest, it is not even clear they would be interesting at all from a theoretical standpoint; so, we believe that aiming at statistical significance there was not fitting. An alternative approach was to settle on an effect size that would be large enough to be of any value. But this is another very difficult issue to tackle: how do we decide what is big enough to be theoretically relevant? Therefore, we eventually decided to reverse our reasoning; we computed the sample size that would be required to reach 90% power in the learning and recognition memory tasks, where we believe that the effects of interest are very likely to be large enough. Using that sample size, we then back-computed what effect size we would be able to address in the congruency and definition selection tasks with a 90% power.

For the learning task, the questions that we wanted to address are (i) whether the novel words are learned in the first place, and (ii) whether their learning depends on the type of ending. This translates into a main effect of number of encounters, and an interaction between number of encounters and type of ending. Based on the literature [6,37,38,68], the results of the pilot experiment and the results of the power analysis itself, and in an effort to keep the design and the analysis as simple as possible, we chose to consider gaze duration and total duration. The former would specifically track relatively early processing, while the latter would encompass both early and late processing stages. The correlation between the two dependent variables was 0.43 in the pilot data, which indicates that they do indeed capture different cognitive processes.

For the recognition memory task, the behavioural part of the sentence congruency task and the definition selection task, we wanted to ask whether there is an influence of ending type in their performance, i.e. we are looking for an effect of complexity.

Finally, for the eye movements analyses in the sentence congruency task, we considered gaze duration and total duration, again with the aim of simplifying the design and the analysis as much possible, while still addressing the relevant processing stages.

#### 3.4.2. Results

**Figure 4.**
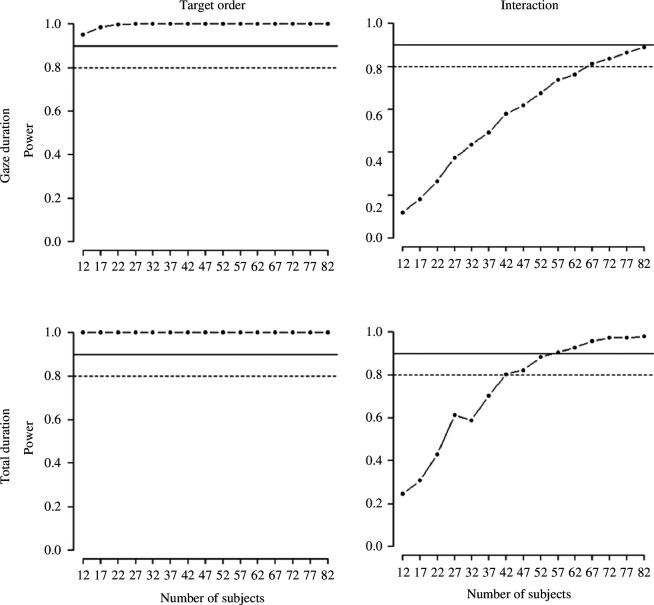
Power analysis for the learning task. Effect of target order and interaction between target order and complexity.

**Figure 5.**
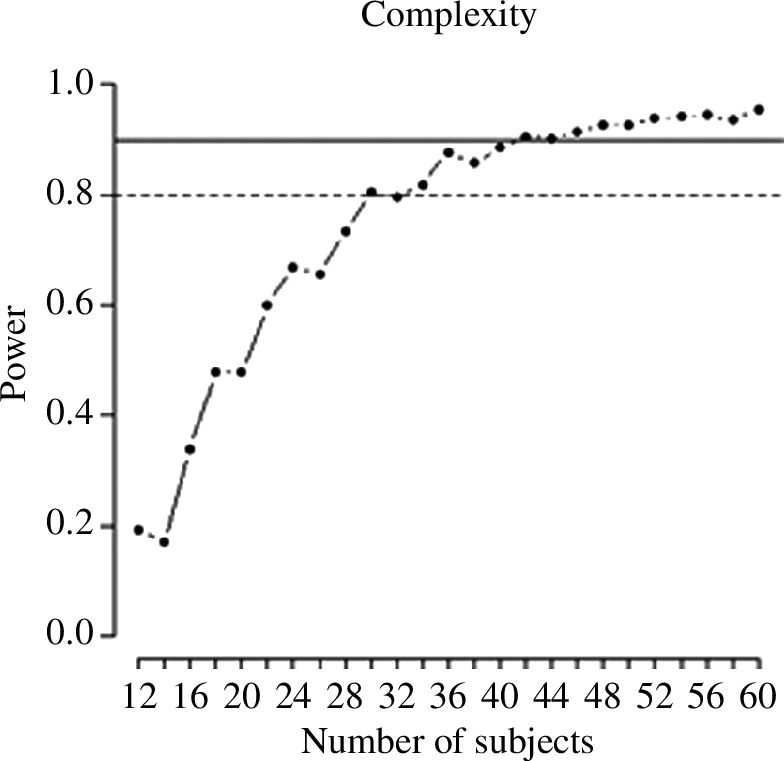
Power analysis for the effect of complexity in the recognition memory task.

The results of the power analysis for the learning task and recognition memory task are illustrated in figures 4 and 5, respectively. The effect that required the most participants is, predictably, the interaction between number of encounters and type of ending in the gaze duration analysis; in order to reach a 90% power here, we needed 84 subjects. This sample size would guarantee an even higher power on all other effects of interest, for all other tasks and dependent variables.

**Figure 6.**
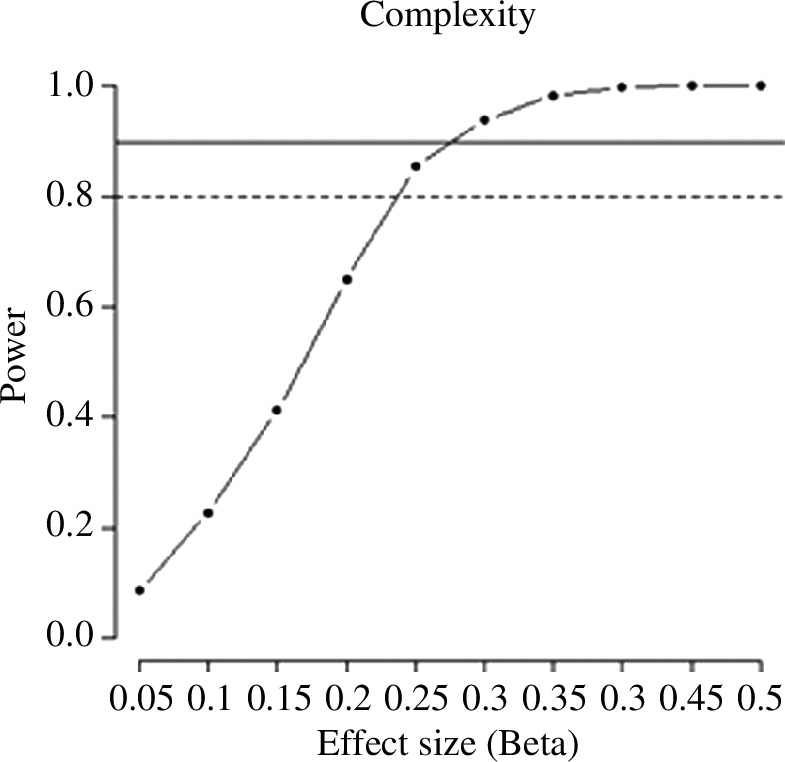
Power analysis for the effect of complexity in the sentence congruency task, as tracked by the explicit judgement.

**Figure 7.**
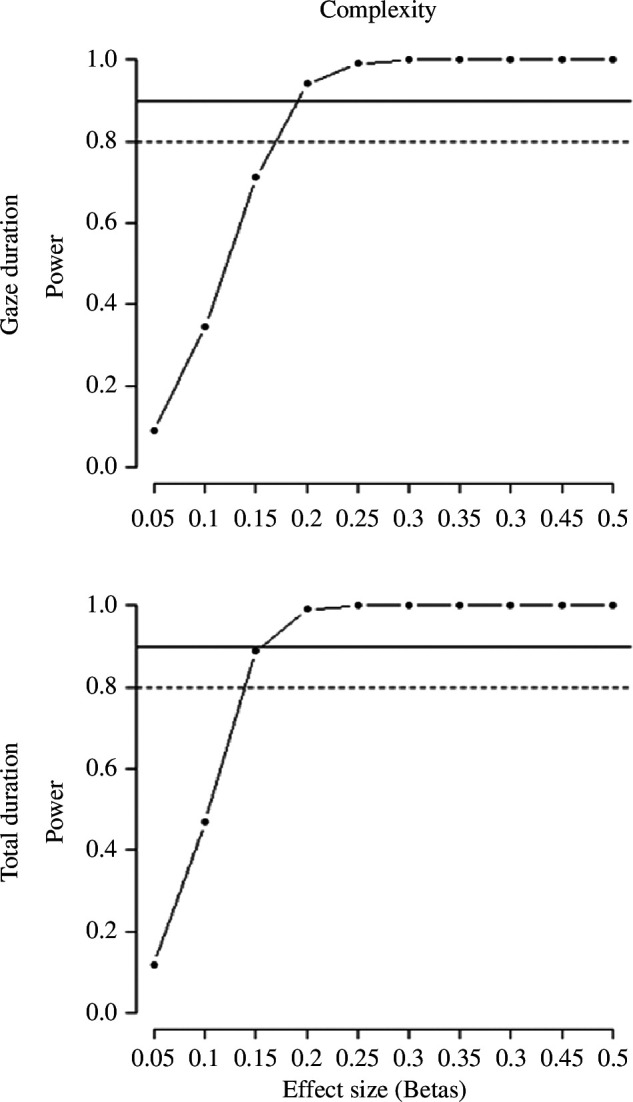
Power analysis for the effect of complexity in the sentence congruency task, as tracked by the gaze duration and total looking time.

**Figure 8.**
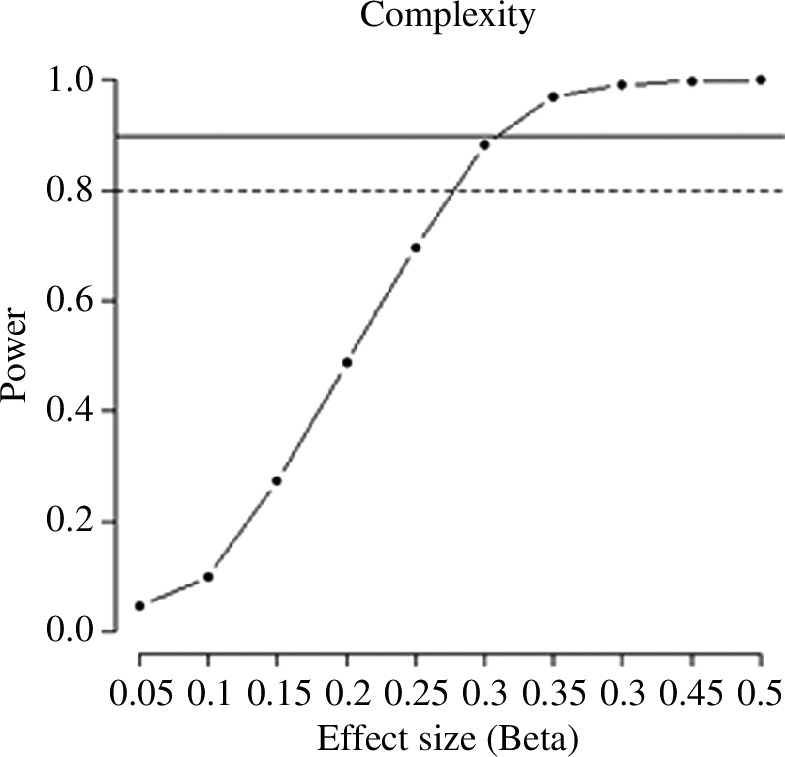
Power analysis for the effect of complexity in the definition selection task.

Considering a sample size of 84 participants and a desired power of 90%, we computed the effect size that would identify as significant in the sentence congruency and the definition selection tasks. For the behavioural part of the sentence congruency task (figure 6), the power analysis showed that we could target an effect size of *β* = 0.28. For the eye tracking part, we were able to target an effect of *β* = 0.19 and *β* = 0.15 for gaze and total duration, respectively (figure 7). Finally, in the definition selection task we could detect an effect size of *β* = 0.31 (figure 8).

### 3.5. Revised analysis plan

To sum up, in consideration of the pilot experiment and the power analyses illustrated above, this is the revised data analysis plan. Unless stated otherwise, everything remained as described in the §2, and the reader can refer to the analysis script of the pilot data for reference.

For the learning routine, we analysed gaze duration and total looking times as a function of complexity (suffixed novel words versus high-frequency ending novel words versus low-frequency ending novel words), number of encounters and their interaction. We expected the eye tracking metrics to shrink with more encounters with the novel words; this was interpreted as a sign of word learning. If morphology has a role in this process, we expected the eye tracking metrics to reduce more with number of encounters in suffixed condition compared with the other two. If learning is driven more by letter frequency instead, the reduction in looking times was expected to emerge to a similar extent with suffixed and high-frequency items, but should be stronger in these conditions than in the low-frequency condition. This task provided an implicit measure of learning and word representation.

In the recognition memory task, we modelled accuracy as a function of complexity. Again, if the presence of a suffix enhances learning, we expected accuracy to be higher in the suffix condition, as compared with high-frequency and low-frequency ending items. If cluster frequency is what makes learning stronger, then we expected suffixed novel words to pattern with high-frequency items, and both conditions should yield higher accuracy than low-frequency ending novel words. This task provided an explicit measure of learning and word representation. To control for potential biases towards a YES or a NO response, particularly given their different number in the current design, we also conducted a *d*-prime analysis, as per the pilot data above. That is, we computed the average *d*-prime by condition, to check that whatever pattern will have emerged in the accuracy analysis above is confirmed with a measure of accuracy that is independent of response bias. This analysis was used descriptively, in support of the main accuracy analysis (hence the lack of a power analysis here).

For the sentence congruency task, we modelled again gaze duration and total looking time as a function of complexity, congruency with the carrier sentence and their interaction. If the participants extract the meaning of the stem from the novel words, they were expected to exhibit longer reading times in the incongruent condition. If the stem is more successfully extracted in the suffixed novel words, the effect of congruency was expected to be stronger for these items. If, however, it is due to the frequency of occurrence, we expected to see a difference between suffixed and high-frequency items on one side, and low-frequency items on the other. This task provided an implicit measure of learning of the stem’s meaning/representation.

In the sentence congruency task, participants also provided a congruency judgement—whether the sentence makes sense or not. This provided a more explicit index of stem learning. The model was the same as above; the accuracy of the congruency judgement was modelled as a function of complexity and congruency itself. We only focused on the complexity effect here, and the possible interpretation of the results is the same as above; if stem learning is triggered by morphology, then accuracy should be higher with the suffixed novel words than in the two non-suffixed conditions. If the learning is triggered instead by the frequency of letter chunks, we expected the high-frequency ending items to pattern with the suffixed novel words, and both conditions being better than low-frequency endings.

For the definition selection task, we modelled accuracy as a function of complexity and, again, we expected suffixed items to be better than the non-suffixed conditions, if stem learning is triggered by morphology. We expected instead the suffixed and high-frequency ending conditions to be better than the low-frequency ending condition if the learning of the stem is triggered by the frequency of the final cluster.

Of course, it was also logically possible that some stem learning would happen in all three conditions, without significant differences. In fact, the pilot data suggested that this scenario was not so unlikely. Such pattern would indicate that readers tend to assign meaning to sub-lexical chunks independently of the structure of the novel words—or at least, independently of the factors we manipulated here, that is, letter chunk frequency and meaning.

As an exploratory analysis, we also investigated whether the morphological effects that emerged in the analyses described above correlate with each individual participant’s MIF obtained from the morpheme interference task. We performed this analysis by using the MIF as a further fixed effect in the models described above.

### 3.6. Modifications of the materials upon revision

**Figure 9.**
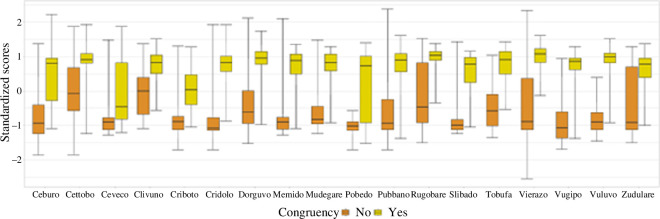
Rating scores across congruency for each novel word, as represented by boxplots. Scores were standardized within subjects, so that we got rid of any effect related to a different use of the scale (some participants tended to give higher scores than others, as one might expect).

Upon a reviewer’s suggestion, we decided to validate the sentence congruency manipulation through a questionnaire. We created two surveys, one for each rotation, so that the raters would see each novel word only once, either in the congruent or the incongruent sentence (see §2.3.2). We asked the respondents to assign ratings from 1 (completely implausible) to 10 (completely plausible). A definition of each novel word was provided with each sentence. We collected data from 41 native Italian speakers, 21 for rotation 1 and 20 for rotation 2. The standardized scores are illustrated in figure 9, and are overall very encouraging; what we designed as congruent sentences were rated much higher in plausibility than what we designed as incongruent sentences (1Q: 0.48 versus −0.79; median: 0.63 versus −0.67; 3Q: 0.75 versus −0.45; mean: 0.5, s.d. = 0.26 versus −0.59, s.d. = 0.28). However, the ratings were quite close in the congruent and incongruent conditions for some novel words. As a cut-off metric, we took the difference between the medians in the congruent and incongruent sentences. There were four items for which this metric was below 1: *cettobo,* −0.97; *ceveco,* −0.43; *clivuno,* −0.82; and *criboto,* −0.92. Since the data were standardized within subjects, this cut-off corresponds to a distance of 1 s.d. in each participant’s response. We decided to change these sentences, and collected further ratings with the revised items with a new group of 22 native speakers. The relevant medians differed enough to satisfy our criterion: *cettobo*, −2.15; *ceveco*, −1.53, *clivuno;* −1.84; and *criboto*, −1.04. We therefore administered the revised sentences for these items in the main experiment.

#### 3.6.1. Study design table

To sum up, table 2 illustrates the general questions addressed in the Registered Report, and how they translate into specific hypotheses and analysis plans. Table 2 incorporates the insight we obtained from the pilot study.

**Table 2.** Study design table.

question	hypothesis	sampling plan	analysis plan	rationale for deciding the sensitivity of the test for confirming or disconfirming the hypothesis	interpretation given different outcomes	theory that could be shown wrong by the outcomes
does the presence of a suffix or a high-frequency word ending (versus a low-frequency word ending) modulate the (implicit and explicit) learning of a novel word?	if morphology facilitates word-learning, suffixed items will have higher accuracy in the recognition memory task compared with the high-frequency and low-frequency endings (explicit learning). This might also translate into an effect on implicit learning; if so, gaze duration and total looking time for suffixed items will shorten more across subsequent encounters with the novel words as compared with the other conditions. If, instead, better learning is induced only by the frequency of letter clusters, then suffixed words and high-frequency ending words will pattern together, and differ from low-frequency ending words.	we will use null hypothesis significance testing. We determined sample size via power analysis and obtained an estimate of effect size in a pilot study.	we will use (generalized) linear mixed-effect models with the following structure. Learning task: model←lmer( DV~complexity*target_order+(1|sbj_id)+(1|target),data) Recognition memory task: model←glmer(DV~target_complexity+(1|sbj_id)+(1|target), family = ‘binomial’, data). See §§2 and 3.4 for details. The analysis pipeline is completely specified in the OSF repository.	pilot data and power analysis.	see above in the table. At the most general level, if suffixed words contrast with high- and low-frequency ending words, we will attribute the experimental effects to a genuine effect of morphology. If instead suffixed and high-frequency ending words will pattern and contrast with low-frequency ending words, we will draw the conclusion that letter chunk frequency is the main driver of the effects.	there is no general theory of word learning that we are trying to specifically address here. Rather, we want to start establishing the role of morphemes in the processing, with a particular focus on meaningfulness versus letter chunk frequency.
does the presence of a suffix or a high-frequency word ending (versus a low-frequency word ending) trigger meaning attribution to a novel stem?	stem meaning extraction should happen specifically for suffixed words, if it is driven by morphology, or for suffixed and high-frequency ending words, if it is driven instead by letter frequency. This will be measured through: (i) accuracy in the sentence congruency task; (ii) gaze duration in the sentence congruency task; (iii) total looking times in the sentence congruency task; and (iv) accuracy in the definition selection task.	we will use null hypothesis significance testing. We determined sample size via power analysis and obtained an estimate of effect size in a pilot study.	we will use (generalized) linear mixed-effect models with the following structure. sentence congruency task: (i) Behavioural: model←glmer(DV~complexity_learned_word*congruency+(1|sbj_id)+(1|sentence),family=‘binomial’,data); (ii) Eye tracking: m_tot←lmer(DV~complexity_learned_word*congruency+1|sbj_id)+(1|baseword), data). Definition selection task: model←glmer(DV~complexity_learned_word+(1|sbj_id),family=‘binomial’,data). See §2 and §3.9 for details. The analysis pipeline is completely specified in the OSF repository.	pilot data and power analysis.	see above in the table. At the most general level, if suffixed words contrast with high- and low-frequency ending words, we will attribute the experimental effects to a genuine effect of morphology. If instead suffixed and high-frequency ending words will pattern and contrast with low-frequency ending words, we will draw the conclusion that letter chunk frequency is the main driver of the effects.	there is no general theory of word learning that we’re trying to specifically address here. Rather, we want to start establishing the role of morphemes in the processing, with a particular focus on meaningfulness versus letter chunk frequency.

## 4. Main experiment

### 4.1. Participants

Eighty-five adults (12 male; mean age = 24.36 years, s.d. = 3.05 years) took part in the experiment, one more than required by the power analyses. They were all native speakers of Italian, with normal or corrected-to-normal vision and no reading disabilities. Participants were paid 15 Euros for their participation.

### 4.2. Exclusion criteria

As described in the registered methods, participants for which the *d*-prime was below 1 in the recognition memory task were excluded from any further analysis; they were considered unsuccessful learners. This procedure led to the exclusion of nine participants.

### 4.3. Methods

Testing procedures, apparatus and software were identical to the pilot study, except that we used a desk-mount instead of a tower mount EyeLink 1000 eye tracker.

### 4.4. Registered analyses

#### 4.4.1. Learning task

The novel words were fixated 5.61 times on average, with a relatively small proportion of single fixations (27%). This mirrors the results of the pilot study.

**Figure 10.**
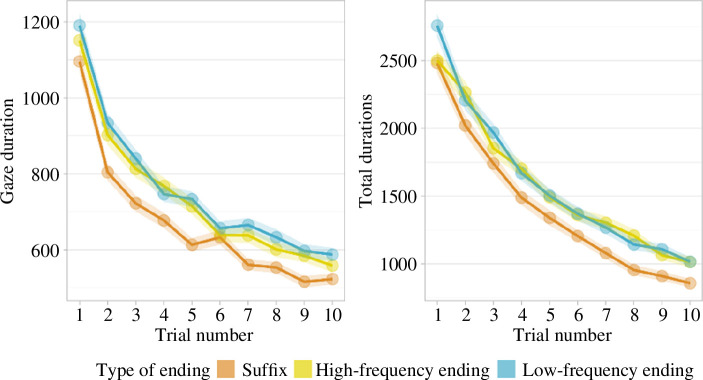
Gaze durations and total durations as they changed during the experiment, across the three complexity conditions. The shadowed areas represent the standard error of the mean.

As per the approved protocol, we ran linear mixed models separately for gaze durations and total durations, with low-frequency endings items set as a baseline. We found a strong influence of number of encounters in both measures (gaze duration: *β* = −0.06, *t* = −22.06, CI = [−0.07 to −0.06], *p* < 0.001; total duration: *β* = −0.11, *t* = −41.04, CI = [−0.12 to −0.11], *p* < 0.001). Moreover, both measures showed that complexity influenced reading, with an advantage for suffixed items compared with both high-frequency and low-frequency endings (gaze durations: *β* = −0.15, *t* = −3.70, CI = [−0.22 to −0.07], *p* < 0.001; total durations: *β* = −0.10, *t* = −2.63, CI = [−0.18 to −0.03], *p* = 0.01). For total duration, a significant interaction emerged between suffixed items and order of presentation (*β* = −0.01, *t* = −2.28, CI = [−0.02 to 0.00], *p* = 0.02); suffixed items had a larger advantage of the order of presentation compared with the other conditions. No such effect surfaced in gaze durations (*β* = 0.00, *t* = 0.71, CI = [−0.01 to 0.01], *p* = 0.48). There was no indication of any difference between high- and low-frequency endings. See figure 10.

#### 4.4.2. Recognition memory task

The mean *d*-prime across participants was 2.34 [−0.66 to 5.7], showing that the participants as a group performed the task very well. However, nine individuals had a *d*-prime lower than 1; as per the approved protocol, we removed them from any further analysis (see §2.8.) which resulted in 75 total participants. The *d*-prime per condition was as followed: suffixed items = 3.19; high-frequency ending items = 2.61; low-frequency ending items = 2.76.

**Figure 11.**
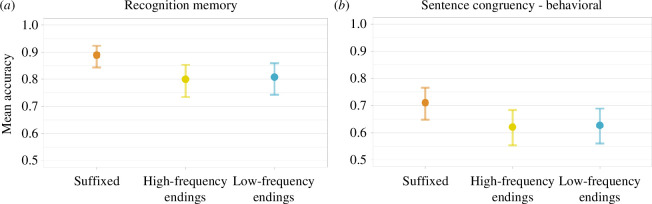
Model estimates for the recognition memory (*a*) and the behavioural data from the sentence congruency task (*b*) across the three complexity conditions. Error bars represent 95% confidence intervals.

The mean overall accuracy was 0.86, confirming that participants learned the novel words successfully. Accuracy was influenced by complexity, with better recognition for suffixed items (mean = 0.88, s.d. = 0.33) as compared with high-frequency (mean = 0.80, s.d. = 0.41) and low-frequency endings items (mean = 0.79, s.d. = 0.40). Generalized linear mixed models confirmed the descriptive results, showing a significant advantage for suffixed items (*β =* 0.61, *z =* 2.34, CI = [0.10 to 1.12], *p =* 0.01); see figure 11 *a*.

#### 4.4.3. Sentence congruency task

##### 4.4.3.1. Behavioural

The overall accuracy in the behavioural task was 0.64. This represents a fairly good performance, which is significantly different from chance in the low-frequency condition (*β* = 0.4, *t* = 2.02, *p* = 0.04) and in the suffix condition (*β* = 0.58, s.e. = 0.2, *z* = 2.9, *p* = 0.004), and very close to significance in the high-frequency condition (*β* = 0.37, s.e. = 0.2, *z* = 1.85, *p* = 0.06).^4^ However, it is also considerably worse than the recognition memory task, which indicates that word forms were learned much better than the meaning of their stems.

Despite some numerical differences between the three conditions (SUFF: mean = 0.69, s.d. = 0.46; HF: mean = 0.61, s.d. = 0.49; LF: mean = 0.61, s.d. = 0.49), generalized mixed-effects model failed to show any significant difference (all *p*s > 0.1; see figure 11*b*).

##### 4.4.3.2. Eye tracking

The average number of fixations was 5.33, with a proportion of refixations 0.7, similarly to the pilot study, probably reflecting an effort to assign a meaning to the novel word.

**Figure 12.**
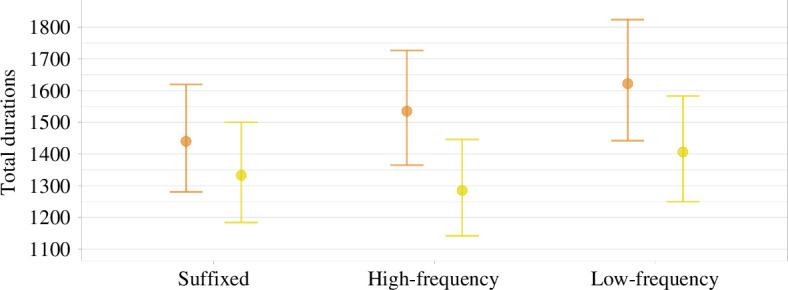
Model estimate for total duration times in the sentence congruency task across the three complexity conditions and two congruency conditions. Error bars represent 95% confidence intervals.

Results showed no significant effect for gaze durations (all *p*s > 0.1). However, an effect of complexity emerged as shorter total duration times for the suffixed items (*β =* −0.12, *t =* −2.32, CI = [−0.22 to −0.02], *p <* 0.02), as compared with high- and low-frequency endings. More importantly, congruent sentences required less reading time (*β =* −0.14, *t =* −2.76, CI = [−0.24 to −0.04], *p <* 0.01; see figure 12). However, congruency did not interact with complexity (*β =* 0.06, *t* = 0.90, CI = [−0.08 to 0.21], *p* = 0.4).

### 4.4.4. Definition selection task

**Figure 13.**
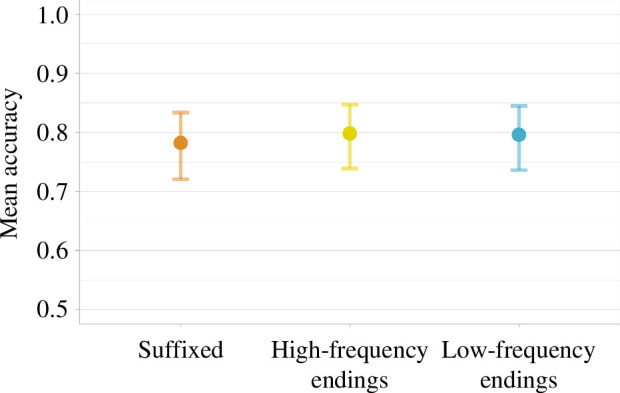
Model estimate for the definition selection task across the three complexity conditions. Error bars represent 95% confidence intervals.

Overall accuracy was 0.75, indicating that participants as a group were able to assign the correct definition to the base word. Replicating the pattern from the pilot study, the complexity of the novel word in the learning phase does not seem to have affected performance: participants picked the correct definition 74% of the times (s.d. = 0.44) for base words in the suffix condition, and 76% of the times for base words in the high- and low-frequency ending condition (s.d. = 0.43 and 0.43, respectively). The linear mixed-effects model confirms this pattern (all *p*s > 0.1; see figure 13).

### 4.5. Exploratory analysis—sensitivity to morphemes

As indicated in the §3.5, we ran exploratory analyses to check for the influence of sensitivity to morphemes in the tasks where effects of complexity were found. This is achieved by incorporating the MIF in the relevant models, as a measure of participants’ sensitivity to the presence of morphemes in a non-word.

#### 4.5.1. Learning task

**Figure 14.**
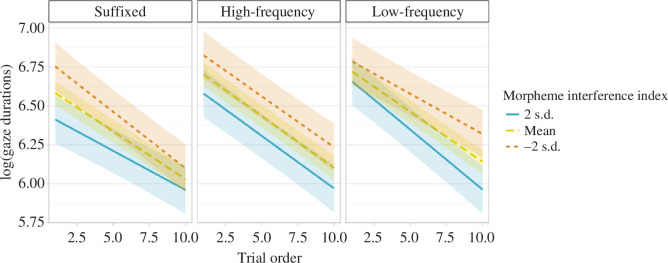
Predicted gaze duration as a function of the interaction of the order of presentation and morpheme interference index. The shaded area is the 95% confidence interval.

The MIF did not influence either gaze or total durations as a fixed effect. However, MIF interacted significantly with trial order (gaze durations: *β =* −0.01, *t =* −2.18, CI = [−0.01 to 0.00], *p <* 0.03; total durations: *β =* −0.01, *t =* −2.33, CI = [−0.01 to 0.00], *p <* 0.02); a larger gain in reading time across multiple encounters with the novel word was associated with stronger sensitivity to morphology. Most importantly, suffixed items specifically interacted with MIF in gaze durations (*β =* −0.05, *t =* −2.56, CI = [−0.09 to 0.01], *p <* 0.01), with more advantage in the suffixed condition for participants who are more sensitive to morphology. Additionally, suffixed items were involved in a three-way interaction, involving the order of presentation and MIF (*β =* 0.01, *t =* 2.90, CI = [0.00 to 0.02], *p <* 0.004). Participants with higher sensitivity to morphemes displayed lesser learning across multiple encounters with the novel suffixed words, although this is probably a side effect of these participants starting off with shorter gaze durations upon their first encounter (see figure 14, leftmost panel).

#### 4.5.2. Recognition memory task

The generalized linear mixed-effect model applied to accuracy data revealed no interactions between sensitivity to morphology and complexity (all *p*s > 0.1).]

#### 4.5.3. Sentence congruency task—eye tracking

The generalized linear mixed models ran on total durations revealed no interactions between morphology and either complexity or congruency (all *p*s > 0.1).

## 5. Discussion

The goal of the present experiment was to explore the role of affixes in novel word learning during sentence reading. To this aim, we presented participants with novel words composed of a novel stem and a familiar affix (e.g. *flib-er*). These items were contrasted with novel words featuring non-meaningful endings that were either matched in frequency with the suffixes (e.g. *flib-an*) or much less frequent (e.g. *flib-ov*). In this way, we were able to separate the role of morphemes as meaning-bearing units versus chunks of letters that frequently co-occur (i.e. that are statistically associated). We assessed both participants’ memory for the novel word itself and their ability to assign meaning to the novel stem, both implicitly (e.g. via eye tracking) and explicitly (e.g. via recognition memory).

In the first task of our experimental paradigm (the learning task), participants encountered each new word in 10 different sentences, while their eye movements were monitored. We found that both gaze durations and total looking times decrease with every subsequent encounter with the novel word. This indicates that processing became increasingly less effortful, suggesting that participants were more familiar with the items to be learned. This is in line with the results of the pilot study and confirms that the paradigm was successful and did trigger word learning (see also [3,6,38]). Suffixed items were consistently read more quickly throughout the entire task, both in gaze durations and total looking time, probably reflecting a relatively higher familiarity with the encounter of a novel stem attached to a familiar affix, as compared with non-affixal word endings. These findings replicate the pattern found by Ginestet *et al.* [37], who also found reduced gaze durations and total durations for complex pseudo-words compared with their orthographic counterparts. The frequency of the endings does not seem to play any relevant role here.

Most importantly, in total reading times, word type interacted with number of encounters, reflecting a more substantial gain in processing speed for suffixed compared with non-suffixed items. In other words, not only did suffixed words lead to shorter fixations overall, but they also yielded a stronger learning effect. Again, the frequency of the novel word ending does not seem to play any particular role here. This interaction only emerged in total looking times, not in gaze duration. This indicates that the effect is probably driven by refixations: apparently, readers have less of a need to revisit unfamiliar words when they are suffixed. This could be explained both by orthographic familiarity—the novel item looks more natural—or by semantics—readers were able to attribute meaning to the novel item, at least to some extent, already during the first pass. We will expand upon this in the subsequent sections when we discuss the results of the novel stem tasks.

The results of the recognition memory task confirm the main findings illustrated above: suffixed items were easier to remember and recognize as compared with non-suffixed words, and the frequency of the word ending did not appear to have a significant impact. Therefore, the learning pattern appears to be the same, independently of whether it was tested implicitly (i.e. via eye tracking) or explicitly (via recognition memory). Note that the distractors contained also recombinant items, i.e. combinations of stems and endings that both appeared in the learning task, but not together; this made it impossible for the participants to perform the task based on the identification of single word parts. Importantly, the present results replicate Tamminen *et al.*’s [34] results, whose recognition memory task served as a model for ours. In their experiment, Tamminen *et al.* found that novel complex words (which in their experiment consisted of an existing stem and a novel affix) were successfully recognized after training. However, their study showed no immediate post-training effects in the measures of implicit learning. One important difference here is that we tested for implicit learning *during* training, while Tamminen *et al.* administered their implicit learning task *after* training, as is the case in other word-learning studies tapping into explicit/implicit representations [42,75]. We do not see any obvious reason why this might have caused inconsistent results; yet, it is an important procedural difference, which we thought was important to mention.

Our findings are congruent with the general consensus that affixes are easily identifiable within a word and constitute a critical processing unit [13,18,19,21,57,76]. These results are also in line with the abundant evidence showing that affixes play a critical role even in non-words [23,25,27,29–32]. We go beyond these findings by showing that suffixes play a prominent role also during the word-learning process.

We also showed that the morphological advantage we uncovered here is due to the fact that affixes are meaning-bearing units; frequency of occurrence does not seem to play any role. On the one hand, this is consistent with previous findings that non-words with suffixes are more easily assigned meaning compared with simple non-words [36,77]. On the other hand, however, the finding that high-frequency endings did not exhibit any similarity with the suffixed items is at odds with the general pattern found in masked priming studies, where pseudo-complex words (e.g. *corner*) still display morphological effects [39,40,78] and facilitates the identification of their pseudo-stems (e.g. *corn*) similarly to real suffixes. Moreover, Lelokiewicz *et al*. [41] demonstrated that readers automatically identify chunks of unfamiliar pseudo-letters (i.e. with no connection to orthography, phonology or meaning) that resemble suffixes in their statistical features, indicating the significance of regularities in letters co-occurrence.

A fairly straightforward explanation for these discrepancies lies in the nature of the tasks employed. Our study focused on the learning of the meaning of the novel words (e.g. they were presented in the context of otherwise familiar sentences), while masked priming studies primarily address early processing stages that rely heavily on the visual characteristics of words rather than their semantics. Along the same lines, Lelokiewicz *et al*. [41] deprived the input of any linguistic information: it is only natural that learners would rely on the only information available—in this case, the statistics of the visual symbols.

In addition to learning the novel words themselves, our participants used the contextual information provided by the carrier words and sentences to infer the meaning of the novel stem. This is the main novelty of our paradigm. In contrast to previous work that focused predominantly on the learning of novel words with a morphological structure [37], our study also evaluates whether participants actually attribute meaning to the novel stems. Here, we show that this is the case in three ways. Firstly, at the explicit level, readers were able to: (i) distinguish sentences that were congruent with the meaning of the stem from sentences that were not; and (ii) to identify the correct definition of the stem among distractors. These effects appear to be smaller in size than for the whole-word learning (although it is quite difficult to make a direct comparison due to the different nature of the tasks and measures); nonetheless, they are clearly statistically significant. Secondly, at the implicit level, novel stems embedded in sentences whose meaning was incongruent with that of the stem tended to elicit longer fixations. Thus, it appears that the process of meaning assignment to the novel stems was deep enough to emerge both when participants’ intuitions were probed directly with an explicit question (e.g. Does this sentence make sense?) and in a kind of behaviour that leaves little access to conscious, strategic control (eye movements during reading).

Interestingly, the eye movement effect only emerged in total looking times; during first-pass reading (i.e. in gaze duration), congruent and incongruent sentences did not trigger any different visual exploration of the text. This suggests that during the first pass, visual/orthographic familiarity with the stem was the main driver of readers’ behaviour, while semantic integration with the sentence context only happened later in time, when participants’ eyes had already moved forward to the text following the critical word. Of course, this does not necessarily generalize to everyday reading. This result might be fully dependent on the fact that our participants were only starting to acquire the novel words, which they encountered only 10 times in total during the learning phase of the experiment. While this is a solid number for a controlled, laboratory-based experiment such as ours (for a similar design, see [6,37,38,68]), it is still far from any realistic exposure in everyday life. This might be the reason why meaning integration was slow in the present experiment—quite possibly slower than during the reading of entirely familiar sentences.

Another critical aspect of these stem meaning effects is that they do not seem to be specifically connected to morphology. In fact, we should probably not speak of stem meaning entirely, but rather, of some more general attribution of meaning to word parts. This is shown by the lack of statistically significant difference between suffixed, high- and low-frequency words in the eye tracking measures, as well as in the explicit judgements on sentence congruency and in the definition selection task. Thus, quite interestingly, there was no need for the presence of an affix in order for the readers to assign meaning to word parts. As we discussed in the registered protocol, before any data were collected, this finding is very relevant theoretically. It reveals that readers would assume a general correspondence between orthography and semantics, such that if, e.g. *fliber*, *fliban* or *flibov* have a meaning related to food, then *flib* must too—independently of the fact that *-er* is an affix, while *-an* and *-ov* are not, and also independently of the fact that *-er* and *-an* are very frequent word endings, while *-ov* is not. It appears that when we encounter a novel word, the process of semantic generalization is not specifically driven by the identification of sub-lexical chunks with a clear meaning on their own. Instead, it is more likely that readers assume a broader, though possibly less precise, correspondence between a word’s form and its meaning, one that does not require a discretized analysis of lexical items or the rule-based combinatorial computations often implied by morphological theory. These findings align more closely with work and theories placing morphology in the wider context of linguistic regularities [11,12,72,79]. In this context, the brain actively searches for any probabilistic relationships between form and meaning, and then leverages this information to interpret novel input.

Of course, this pattern of results might potentially be contingent on the paradigm we used here. For example, as a reviewer suggested, there is the possibility that since participants were exposed to many items that indeed contained an existing suffix, they were encouraged to segment all items, even in the absence of morphology. This is certainly possible, and there is no doubt that more work—possibly using a diversity of experimental paradigms—is necessary before we can hold these results as a general phenomenon in word learning. On the other hand, a third of the novel words that the participants learned here included a suffix, which is well below the estimated percentage of morphologically complex words in some languages (e.g. 80% in English [79]). Moreover, even if this sort of ‘overgeneralization’ of word compositionality (or semantic transparency, at least) was boosted by the specific paradigm used here, this would still show that readers are not bound by the presence of a suffix (or a stem) and, at least when encouraged by the context, tend to disregard the fundamental tenet of symbolic systems (form is arbitrarily associated with meaning) and impose some form of semantic relatedness between orthographically similar items.

The individual variability data seem to support this view, at least in part. In the exploratory section of the analyses, we examined whether readers’ sensitivity to morphology, as assessed using a morpheme interference task, could explain their learning patterns—either of the whole words or of the stems’ meaning. We did not observe any effect for what concerns the latter: the size of each participant’s morpheme interference effect does not seem to correlate with their visual exploration of the novel stems in congruent sentences compared with incongruent sentences. Similarly, it did not correlate with their ability to explicitly discern if a sentence is meaningful or nonsensical, or to identify the definition of the novel stems.

On the other hand, we did obtain a morpheme interference effect in the learning task. We would tend to discard the highest level, three-way interaction as quite irrelevant theoretically. In the suffixed condition, there seems to be a *lesser* learning effect for those individuals with *higher* sensitivity to morphemes (see figure 14). This is clearly theoretically implausible. Indeed, the effect might be explained by the fact that suffixed words attracted relatively quick fixations already at the beginning of the learning routine in individuals with strong morpheme interference effects. As a consequence, there would simply be less room for improvement compared with people less sensitive to morphemes.

We tend to trust more the two-way interactions, because their statistical power was certainly higher and because they depict a more coherent theoretical message. People with a stronger morpheme interference effect generally displayed stronger learning *across conditions*; and also, they also showed a larger advantage for suffixed items *overall*, *across successive encounters with the novel words*. This latter result suggests that the morpheme interference effect might capture some specific morphological sensitivity independent of the learning effect shown in the present study. Conversely, the former finding, points again to some general learning ability, which might be reflected in the morpheme sensitivity score, but, again, cuts across conditions in the present experiment—that is, does not seem to be specific for affixes.

In summary, the present study revealed that suffixes significantly contribute to novel word learning. This influence is driven by their semantic component, not by the statistical associations between letters that morphology implies. Furthermore, this effect emerged in both explicit and implicit tasks. Importantly, readers seem to generalize the meaning of the whole word to its constituent parts, but this process is not specifically triggered by the presence of an affix. Instead, it seems to reflect a more general attempt of the brain to identify regularities in the mapping between form and meaning.

## Data Availability

The full set of materials, data and analysis scripts isis available on OSFat [80]. It contains the stimuli, the pilot data, the main experiment data and the analyses.

## Footnotes

^1^
Except for a few loan words (e.g. brioche, chef, élite) and a small set of accented items (e.g. caffé).

^2^
We did not carry out null hypothesis significance testing with the pilot data described below, given that it would obviously be heavily underpowered. Even more importantly, assessing statistical significance is not the goal of the pilot experiment.

^3^
Because accuracy models were estimated using a logistic link function, these numbers are expressed in log odds.

^4^
These betas refer to the intercept term. The logistic link function implies that 0 on that term represents chance level.
